# Impact of COVID-19 on the mental health of adolescents and youth in Nairobi, Kenya

**DOI:** 10.3389/fpsyt.2023.1209836

**Published:** 2024-02-08

**Authors:** Peter B. Gichangi, Meagan E. Byrne, Mary N. Thiongo, Michael Waithaka, Bianca Devoto, Elizabeth Gummerson, Shannon N. Wood, Philip Anglewicz, Michele R. Decker

**Affiliations:** ^1^International Centre for Reproductive Health Kenya, Mombasa, Kenya; ^2^Department of Environment and Health Sciences, Technical University of Mombasa, Mombasa, Kenya; ^3^Department of Public Health and Primary Care, Faculty of Medicine and Health Sciences, Ghent University, Ghent, Belgium; ^4^Department of Population, Family and Reproductive Health, Johns Hopkins Bloomberg School of Public Health, Baltimore, MD, United States

**Keywords:** adolescents, COVID-19, depression, Kenya, mental health, mixed method, PHQ-2, youth

## Abstract

**Objective:**

To report on the mental health status of adolescents and youth in relation to the COVID-19 pandemic in Nairobi County, Kenya.

**Methodology:**

This was a mixed-methods study with cross-sectional quantitative and qualitative components conducted in Nairobi County, Kenya from August to September 2020. The quantitative survey involved phone interviews of *n* = 1,217 adolescents and youth. Qualitative components included virtual focus group discussions (FGDs) with adolescents and youth (*n* = 64 unmarried youths aged 16–25 years, across 8 FGDs) and youth-serving stakeholders (*n* = 34, across 4 FGDs), key informant interviews (*n* = 12 higher-level stakeholders from Ministries of Health, Gender, and Education), and in-depth interviews with youth (*n* = 20) so as to examine the COVID-19 impact on mental health.

**Results:**

Among the participants, 26.6% of young men and 30.0% of young women reported probable depressive symptoms, of whom 37.7% of young men and 38.9% of young women reported little interest or pleasure in doing various activities. Hopelessness and feeling down nearly every day was additionally reported by 10.7% of young women and 6.3% of young men. Further, about 8.8% of young men and 7.6% of young women reported they could not get the emotional help and support they may need from people in their life. Multivariable regression results showed an association between depressive symptoms and reduced working hours due to COVID-19 and increased intimate partner violence. Additionally, the results show that respondents with higher emotional help and support were less likely to report depressive symptoms. Qualitative results confirm the quantitative findings and exemplify the negative behavior arising from the impact of adherence to COVID-19 prevention measures.

**Conclusion:**

Mental health issues were common among adolescents and youth and may have been augmented by isolation and economic hardships brought about by COVID-19 restrictions. There is a need for concerted efforts to support adolescents and young people to meet their mental health needs, while considering the unique variations by gender. There is need to urgently strengthen the mental health system in Kenya, including via integrating psychosocial support services in communities, schools, and healthcare services, to ensure adolescents and young persons are not left behind.

## Introduction

Mental health is defined as “a state of well-being in which every individual realizes his or her own potential, can cope with the normal stresses of life, can work productively and fruitfully, and is able to make a contribution to her or his community” (1). In 2018, it was estimated that about 10.7% of the global population, or 792 million people worldwide, were living with a mental or behavioral disorder; 264 million, or 3.4% (2–6%), suffered from depression (2). Mental health disorders are the leading cause of years lived with disability (YLDs) (3, 4), accounting for one in every six YLDs globally (3, 4). Mental health disorders present in both sexes, all age groups, and across all regions of the world (4).

Adolescents are a high priority group for assessing and treating mental health disorders given age at onset and impact on health trajectories. It is estimated that about half of all mental health disorders in adulthood start by age 14 (5). Approximately 14% of the world’s adolescents aged 10–19 years live with a mental health disorder (4), and such disorders are a leading cause of disability in young people globally (4, 6, 7). In 2021, it was estimated that one in seven (14%) 10–19-year-olds experienced a mental health condition, most of which are unrecognized and untreated. Adolescents with mental health conditions are particularly vulnerable to social exclusion, discrimination, stigma, educational difficulties, risk-taking behaviors, physical ill-health, and human rights violations (4).

Following a life course framework, adolescents’ mental health impacts their subsequent mental health and general health as adults. Mental health conditions can have a substantial effect on all areas of life, including school or work performance, relationships with family and friends, and ability to participate in the community (4). Mental health conditions among adolescents can have long lasting negative impacts not only at the personal level, but at the national level in terms of treatment and lost productivity (8). Two of the most common mental health conditions, depression and anxiety, cost the global economy US$ 1 trillion each year (4). Despite these figures, the global median of government health expenditure that goes to mental health is less than 2% (4).

On top of the existing burden of mental health conditions globally, mental health status is likely to be impacted negatively by COVID-19 pandemic, as has been shown in previous pandemics and epidemics (9, 10). The advent of the COVID-19 pandemic brought untold disruptions in health and education, confinement to homes, social isolation, and economic meltdown at the individual, community and national levels (10). Epidemics accentuate or create new stressors, including fear and worry for self or loved ones, constraints on physical movement and social activities due to quarantine, and sudden and radical lifestyle changes (3). Recent reviews of outbreaks and pandemics have found that stressors, such as infection fears, frustration, boredom, inadequate supplies, inadequate information, financial loss, and stigma led to some form of mental health challenges (9, 10).

There are studies showing the impact of COVID-19 on children’s, adolescents’, and youths’ mental health status. These studies have shown that compliance with COVID-19 restrictions and social isolation were significant challenges for children and adolescents, in turn causing stress that can lead to mental health problems (14–17). The 2021 study among children and adolescents in Canada reported that stress from social isolation, from both the cancellation of important events, such as graduation ceremonies, school trips, vacations, and the loss of in-person social interactions, was strongly associated with deterioration in mental health across all domains (14). Another study during the onset of COVID-19 in Shaanxi Province, China among children and adolescents found that 32% had irritability and 28% showed worry (18). A study measuring the immediate psychological effects of the COVID-19 quarantine among children and adolescents 3–18 years in Spain and Italy reported the most frequent symptoms were difficulty concentrating, boredom, irritability, nervousness and worry (19). Further, the study by Son et al. among college students in USA found 71% of students reported increased stress and anxiety due to the COVID-19 outbreak (20) and Novotny et al. showed that the prevalence of moderate to high stress and depressive symptoms increased 1.4- and 5.5-fold, respectively, during the COVID-19 lockdown (21).

Prior to the onset of the COVID-19 pandemic mental health services were sparse in low- and middle-income countries (LMICs), such as Kenya. Moreover, the burden of mental health for adolescents was unclear given the lack of national data on this topic. COVID-19’s potential to accentuate or trigger mental health challenges in countries where mental health services are not optimal, like in sub-Saharan Africa, has raised concern. Most studies examining mental health stressors related to COVID-19 are from developed countries or the earliest hotspots in China (22). While there is widespread acknowledgment of the possible impacts of COVID-19 on mental health, there is lack of appropriate data among adolescents and youth in Kenya or similar contexts. In Kenya, between 13th March and 31st July 2020, new guidance was created to address mental health during the pandemic, including The National Readiness and Early Response Plan to Mental Health Education and the National Disaster Response Plan (23). While the mental health of adolescents are addressed in some of these national priorities, not all aspects of mental health were covered, and implementation was a challenge due to the poorly resourced mental health system (23). In this paper, we report mental health status of adolescents and youth in relation to COVID-19 pandemic in Nairobi County, Kenya.

## Materials and methods

### Study design and population

This was a cross-sectional secondary analysis of mixed-methods (quantitative and qualitative) data collected in Nairobi County, Kenya from August to September 2020. The parent study, the Nairobi Youth Respondent Driven Sampling Survey (YRDSS), is an ongoing cohort study of adolescents and young adults in Nairobi, Kenya. The parent study aimed to examine youth contraceptive behaviors; at the onset of the COVID-19 pandemic, additional aims were added to understand gendered impacts of the pandemic on youth health and safety. The YRDSS uses respondent-driven sampling (RDS), a sampling method designed to recruit harder-to-reach populations, such as urban youth. RDS is a chain-based recruitment method that begins with purposefully selected seeds, followed by monitored peer-to-peer coupon distribution (24). At baseline (conducted June–August 2019), youth were eligible for cohort participation if aged 15–24, unmarried, and residing in Nairobi County for at least 1 year prior to the baseline survey. During the baseline survey, participants consented to be re-contacted and provided a contact phone number. Further methodological details can be found elsewhere (25, 26).

The 2020 quantitative component was a phone-based follow-up interview of adolescents and youth who participated in the baseline YRDSS. The follow-up (2020) quantitative survey was conducted by trained resident enumerators (REs) in two interview sessions to minimize the time burden on participants. The mental health and social support questions come from the second interview session; there was minimal attrition between the two sessions.

The qualitative component included virtual focus group discussions (FGDs) with youth aged 15–24 years and stakeholders from youth-serving community-based organizations (CBOs), key informant interviews with health care officials and other government officers managing/providing adolescent and youth sexual and reproductive health (SRH) services, and in-depth interviews (IDIs) among a subset of follow-up study participants (adolescents and young adults).

### Quantitative measures

#### Dependent variable: mental health status

The Patient Health Questionnaire-2 (PHQ-2) (27, 28), a pre-screening tool which has been validated in sub-Saharan Africa (29), including Kenya (30), was used to assess presence of depressive symptoms. It consists of the first two items of the PHQ-9 (which assess the frequency of depressed mood and anhedonia). It can be used as a first step to identify patients for evaluation with the full PHQ-9 (31). Specifically, participants were asked about having little interest or pleasure in doing things and feeling down, depressed, or hopeless in the past 2 weeks with 4-response Likert scale, 0-Not at all; 1-Several days; 2-More than half the days and 3-Nearly every day. PHQ-2 score of > = 3 cut-off was used for presence or absence of mental health problems (28).

#### Independent variables

##### Social support scale

Participants were asked four questions about social support that young people could access if need be. We asked about three kinds of support systems, based on the brief version of the Medical Outcomes Study Social Support Survey (32): (i) There is someone in my life I can share my joys and sorrows with, (ii) I have someone to count on when things go wrong, and (iii) I can get the emotional help and support I need from people in my life. The responses were: 0-No response, 1-Strongly disagree, 2-Disagree, 3-Neither agree nor disagree, 4-Agree and 5-Strongly agree. The mean score (sum of all three items, then divided by 3) was computed as the index for social support. An additional question “Who are the people you rely on most for emotional help and support?” was used to capture the persons the adolescents would rely on.

##### Scale of pandemic-related difficulties

Decisional autonomy was assessed using best practices (33); using a six-point Likert scale: 0-No response 1-None; 2-Very little; 3-Some; 4-Fair amount and 5-Full control. For young women, past-year experience of intimate partner violence and non-partner sexual violence were assessed via best practices for violence research.

##### Financial and schooling situation

Using measures developed specifically for the COVID-19 pandemic, participants were asked whether there were changes in their: (i) usual work, (ii) income generation activities and (iii) usual school activities since the advent of COVID-19 pandemic. Inability to meet basic needs since COVID-19 restriction was assessed via 4-response Likert scale dichotomized as 1 = Not very able/Not able at all and 0 = Very able/Somewhat able.

##### Demographic variables

Participants reported on age, sex, highest level of education attended, marital status, household wealth, living situation, and main activity before COVID-19 and their sub-county of residence.

### Qualitative component participants

The qualitative component consisted of FGDs with youth (*n* = 64 unmarried youths aged 16–25 years, across 8 FGDs); FGDs with youth-serving stakeholders (*n* = 34, across 4 FGDs); key informant interviews (*n* = 12 comprising higher-level stakeholders from Ministries of Health, Gender, and Education); and in-depth interviews with adolescent and youth cohort participants (*n* = 20). Youth participants’ recruitment was facilitated by CBO leaders. Coordinators working with the youths and stakeholders were identified via community-partnered recruitment through the assistance of local youth organizations. Using a semi-structured guide, the discussion topics included: risk perception, preventive behavior, contraceptive access and needs, household dynamics, income and educational impact, and safety concerns. Discussions were audio-recorded, transcribed verbatim, and translated to English language for inductive and deductive thematic analysis.

### Quantitative data analysis

Descriptive statistics (frequencies and percentages) were used to summarize the quantitative data. Following descriptive statistics, bivariate logistic regression assessed the association between the outcome (the mental health status) and each independent variable of interest. Any analysis showing a *p-*value <0.05 moved the variable into a multivariable logistic regression model with 95% Confidence Intervals to determine the multivariate effects of COVID-19-related factors on depressive and anxiety symptoms. Data are weighted to account for the RDS design, post-estimation adjustment based on 2014 KDHS population data (age, sex, education levels), and adjustment for loss to follow-up from the 2019 YRDS Survey. Analyses were conducted using Stata Statistical Software Release 15.

### Qualitative data analysis

For qualitative data analysis, a codebook was deductively developed from the interview and focus group discussion guides— triangulating key topics of interest around sexual activity and relationships; contraceptive dynamics; COVID-related risks and perceptions; and COVID impacts on social support, mental health, safety, time use, economic activities, and autonomy. Dual coding was conducted with transcripts from each qualitative activity to establish intercoder reliability and validate the codebook. Once coding agreement was reached, analysts independently coded the remaining transcripts. Through an iterative process, the analysis team applied the codebook to the qualitative data, identified and discussed emergent themes, and inductively developed new codes based on these themes. Atlas.ti software aided management and organization of analysis.

### Ethical consideration

The protocol was reviewed and approved by the Johns Hopkins Bloomberg School of Public Health Institutional Review Board and Kenyatta National Hospital-University of Nairobi Ethics Research Committee (REF: KNH-ERC/A/182). Interviews were conducted after verbal consent/assent was obtained. Waiver of parental/guardian was obtained for minors. The right of individuals not to participate in the study was also respected.

## Results

### Survey sample characteristics

**Figure 1:**
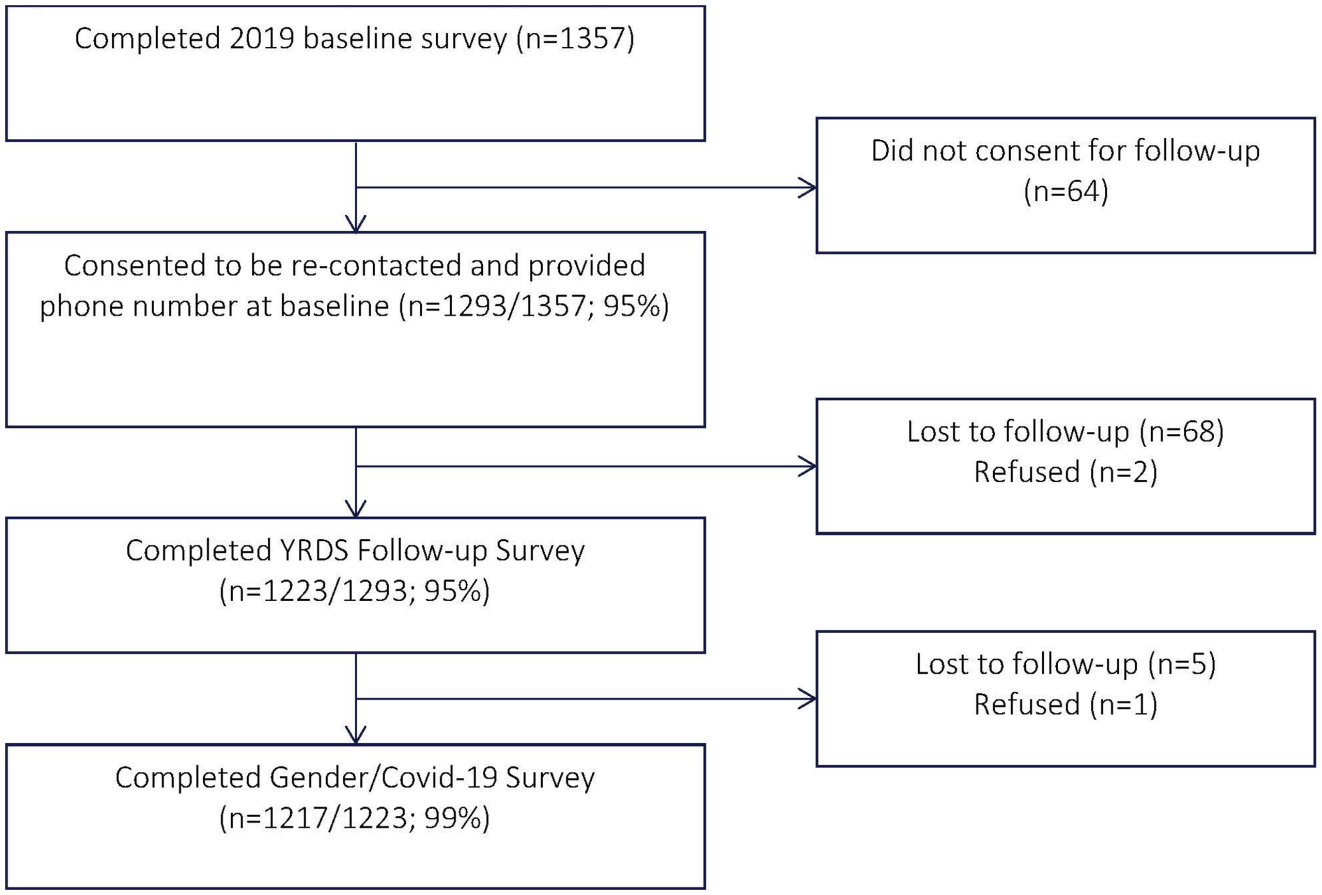
Quantitative component study participant flowchart.

A total of 1,293 adolescents and youth who had consented to be re-contacted and provided a phone number at baseline were approached for participation, of whom 1,217 (94.1%) consented to participate and completed the survey (Figure 1). Of the 1,217 adolescents and young people who participated in the cross-sectional survey, 59.1% were females. The majority of the participants were not married (90.6%), had a secondary education (55.2%), and were living with others (76.6%). Approximately 13.5% of the participants were from the upper half of the wealth ladder and 37.6% were students. They spent more time at home since COVID-19 restrictions (84.9%) and were concerned or very concerned about getting infected with COVID-19 (90.8%). Among the participants who reported living with others, 76.6% reported that the household members spent more time at home since COVID-19 restrictions (Table 1).

**Table 1:** Background characteristics of respondents.

Variable	*N* (unweighted)	*N* (weighted)	% (weighted)
Total	1,217	1,217	100
Sex			
Male	605	498	40.9
Female	612	719	59.1
Age group			
15–19 years	334	333	27.4
20–26 years	883	884	72.6
Highest level of education attended			
Never/Primary	43	95	7.8
Secondary/Post-primary	628	672	55.2
Higher	546	450	37.0
Marital status			
Unmarried	1,115	1,103	90.6
Married	102	114	9.4
Wealth ladder			
Step 1–5	1,059	1,053	86.5
Step 6–10	158	164	13.5
Living situation			
Lives alone	299	284	23.4
Lives with other(s)	918	933	76.6
Main activity before COVID-19			
Student	497	458	37.6
Working in the formal economy	128	128	10.5
Working in the informal economy	346	374	30.8
Other	246	256	21.1
Time spent at home since COVID-19 restrictions			
More time at home	1,067	1,033	84.9
Unchanged/Less time at home	150	184	15.1
Household members’ time at home since COVID-19 restrictions^a^			
More time at home	718	703	76.6
Unchanged/Less time at home	92	112	12.1
Varies by family member	108	103	11.2
Perceived COVID-19 risk			
Concerned/very concerned	1,083	1,105	90.8
Not concerned/ little concerned	134	112	9.2

N, weighted sample; %, weighted percent; ^a^Among participants who reported living with others.

### Mental health status and covariates

Table 2 presents the frequency of core depressive symptoms over the past 2 weeks preceding the survey, as well as social support, decision-making autonomy, intimate partner/sexual violence, and financial situation indicators. Approximately 23.3% of adolescents and youths presented depressive symptoms (21.8% males and 24.3% females). Among young men, 51.2 and 43.0%, respectively, reported having little interest or pleasure in doing things or feeling down, depressed, or hopeless within the past 2 weeks. Among young women, 55.4% had little interest or pleasure in doing things while 54.3% reported feeling down, depressed, or hopeless in the past 2 weeks. About 85% of the young people either agreed or strongly agreed that they could access social support if need be. With regards to decision-making autonomy, almost half (45.4%) of young people reported to have full control over decisions about leaving the house to go into the community, 55.8% reported full control over decisions about seeking health care, while over a third (42.4%) reported having full control over decisions about how to spend money they had earned. About 13% of the young women reported that an intimate partner had violated them physically at least once in the last 12 months; approximately 9% reported being forced to have had sex with a partner and 4% forced to have sex with a non-partner. Six in every 10 young people reported increased changes in their household responsibilities since COVID-19 restrictions.

**Table 2:** Depressive symptoms, and covariates by gender.

Variable	Males – *n* (%)	Females – *n* (%)	Total – *n* (%)
Total	605	612	1,217
Mental health status			
Depressive disorder unlikely	473 (78.2)	463 (75.7)	933 (76.7)
Likely depressive disorder	132 (21.8)	149 (24.3)	284 (23.3)
Little interest or pleasure in doing things			
Not at all	293 (48.5)	273 (44.6)	562 (46.2)
Several days	228 (37.7)	238 (38.8)	467 (38.4)
More than half the days	31 (5.2)	52 (8.5)	87 (7.1)
Nearly every day	52 (8.7)	50 (8.1)	101 (8.3)
Feeling down, depressed or hopeless			
Not at all	345 (57.0)	279 (45.7)	612 (50.3)
Several days	170 (28.0)	208 (33.9)	384 (31.5)
More than half the days	53 (8.7)	60 (9.7)	113 (9.3)
Nearly every day	38 (6.3)	65 (10.7)	108 (8.9)
Social support			
Social support index			
Neutral/Disagree/ Strongly disagree	116 (19.2)	78.3 (12.8)	187.5 (15.4)
Strongly agree/Agree	489 (80.8)	533.7 (87.2)	1029.5 (84.6)
Decision-making autonomy			
How much control do you have over decisions about leaving the house to go into the community?			
Some/None	283 (46.8)	367 (60)	665 (54.6)
Full control	322 (53.2)	245 (40)	552 (45.4)
How much control do you have over decisions about seeking health care?			
Some/None	263 (43.5)	274 (44.7)	538 (44.2)
Full control	342 (56.5)	338 (55.3)	679 (55.8)
How much control do you have over decisions about who you will associate with outside of your household			
Some/None	251 (41.4)	240 (39.3)	489 (40.1)
Full control	355 (58.6)	372 (60.7)	729 (59.9)
How much control do you have over decisions about how to spend money you have earned?			
Some/None	349 (57.6)	439 (71.7)	803 (66.0)
Full control	256 (42.4)	173 (28.3)	414 (34.0)
Intimate partner violence/Sexual violence^£^			
In the past 12 months, has a partner ever pushed you, thrown something at you that could hurt you, punched or slapped you?			
Never		380 (86.7)	
At least once		59 (13.3)	
In the past 12 months, have you had sex with a partner when you did not want to due to threats, pressure, or force?			
Never		400 (91.4)	
At least once		38 (8.6)	
In the past 12 months, have you had sex when you did not want to with anyone else (not a partner) due to threats, pressure or force?			
Never		419 (95.6)	
At least once		19 (4.4)	
Financial situation			
Contributor of the majority of the household income			
Partner/spouse and other contributions	63 (10.4)	131 (21.4)	131 (21.4)
Respondent	322 (53.2)	90 (14.7)	90 (14.7)
Dad or father figure /Mom or mother figure	221 (36.5)	391 (63.9)	391 (63.9)
Changes in the respondents’ household responsibilities since COVID-19 restrictions			
No change	206 (34)	155 (25.4)	352 (28.9)
Decreased somewhat/ a lot	62 (10.2)	60 (9.8)	121 (10)
Increased somewhat/ a lot	337 (55.7)	397 (64.9)	744 (61.1)
How able the respondent is to meet their basic needs since the COVID-19 restrictions			
Somewhat/ very able	333 (55)	285 (46.6)	609 (50.1)
Not very/not at all able	272 (45)	327 (53.4)	608 (49.9)
HH member laid off of work since start of COVID restrictions			
No	540 (89.2)	403 (65.9)	918 (75.5)
Yes	65 (10.8)	209 (34.1)	299 (24.5)
Worked reduced hours since start of COVID restrictions			
No	486 (80.4)	358 (58.5)	821 (67.5)
Yes	119 (19.6)	254 (41.5)	396 (32.5)
Been unable to find work since start of COVID restrictions			
No	519 (85.8)	416 (67.9)	916 (75.2)
Yes	86 (14.2)	196 (32.1)	301 (24.8)
Been unable to work due to illness since start of COVID restrictions			
No	600 (99.1)	584 (95.5)	1,180 (97)
Yes	6 (0.9)	28 (4.5)	37 (3)
Been on unpaid leave since start of COVID restrictions			
No	568 (93.9)	545 (89.1)	1,108 (91)
Yes	37 (6.1)	67 (10.9)	109 (9)
Stopped attending school since start of COVID restrictions			
No	432 (71.4)	285 (46.5)	690 (56.7)
Yes	173 (28.6)	327 (53.5)	527 (43.3)
Gone without meals/ reduced means since start of COVID restrictions			
No	547 (90.5)	473 (77.4)	1,007 (82.7)
Yes	58 (9.5)	139 (22.6)	210 (17.3)

n, weighted sample; %, weighted percent; ^£^Among females who have ever had a sexual partner.

### Bivariate and multivariable analysis

Associations between mental health status (depressive disorder) and background characteristics are shown in Table 3. The main activity of the respondents before COVID-19 was significantly related to depressive symptoms, with those who were working in the formal economy displaying odds of having depressive symptoms compared to students (OR = 2.20; 95% CI: 1.20, 4.03). The odds of having depressive symptoms among those who had someone in their life whom they could share their joys and sorrows with was about 70% lower than those who did not have someone (OR = 0.30; 95% CI: 0.17, 0.52). Similarly, those who had someone to count on when things went wrong and those who get the emotional help and support they may need from people in their life had significantly decreased odds of having depressive symptoms. Respondents with full control over decisions about who they associate with outside of their household had significantly higher odds of having depressive symptoms as compared to those who had no or some control (OR = 1.60; 95% CI: 1.05, 2.44). Respondents who were not very or not at all able to meet their basic needs since the COVID-19 restrictions had higher odds of having depressive symptoms compared to those who were somewhat or very able (OR = 1.85; 95% CI: 1.02, 3.37). Respondents who worked reduced hours since start of COVID restrictions as well as those who had gone without meals/reduced means since start of COVID restrictions were more than two times likely to have depressive symptoms [(OR = 2.45; 95% CI: 1.32, 4.56) and (OR = 2.77; 95% CI: 1.40, 5.49) respectively]. With regards to gender-based violence, young women who had a past-year experience of physical intimate partner violence had significantly higher odds of having depressive symptoms compared to those who had never been violated (OR = 1.96; 95% CI: 1.77, 8.87).

**Table 3:** Bivariate and Multivariable analysis.

	Depressive disorder unlikely	Depressive disorder likely	Bivariate analysis	Multivariable analysis	
	*N* (%)	*N* (%)	Crude Odds Ratio (95% CI)	Among young women only – Adjusted Odds Ratio (95% CI)	Among all young people – Adjusted Odds Ratio (95% CI)
Total	933 (76.7)	284 (23.3)			
Sex					
Male	389 (78.2)	109 (21.8)	Ref.		
Female	544 (75.7)	175 (24.3)	1.15 (0.77, 1.71)		
Age group					
15–19 years	260 (78.2)	73 (21.8)	Ref.		
20–26 years	673 (76.1)	211 (23.9)	1.12 (0.70, 1.80)		
Highest level of education attended					
Never / Primary	62 (64.7)	34 (35.3)	Ref.		
Secondary / Post-primary	523 (77.9)	149 (22.1)	0.52 (0.23, 1.19)		
Higher	348 (77.5)	101 (22.5)	0.53 (0.23, 1.23)		
Marital status					
Unmarried	848 (76.9)	254 (23.1)			
Married	85 (74.4)	29 (25.6)	1.15 (0.61, 2.17)		
Wealth ladder					
Step 1–5	794 (75.4)	259 (24.6)	Ref.		
Step 6–10	139 (84.7)	25 (15.3)	0.55 (0.30, 1.02)		
Living situation					
Lives with other(s)	709 (76)	224 (24)	Ref.		
Lives alone	225 (79.1)	59 (20.9)	0.84 (0.53, 1.33)		
Main activity before COVID-19					
Student	364 (79.5)	94 (20.5)	Ref.	Ref.	Ref.
Formal economy	82 (63.8)	46 (36.2)	2.20 (1.20, 4.03)**	2.26 (1.24, 4.12)**	2.16 (1.19, 3.96)**
Informal economy	276 (73.7)	98 (26.3)	1.38 (0.85, 2.26)	1.41 (0.82, 2.42)	1.51 (0.89, 2.56)
Other	212 (82.6)	45 (17.4)	0.82 (0.47, 1.41)	0.70 (0.40, 1.24)	0.73 (0.41, 1.28)
Time spent at home since COVID-19 restrictions					
More time at home	789 (76.4)	244 (23.6)	Ref.		
Unchanged/Less time at home	144 (78.5)	40 (21.5)	0.89 (0.49, 1.60)		
Household members’ time at home since COVID-19 restrictions^b^					
More time at home	547 (76.6)	167 (23.4)	Ref.		
Unchanged/Less time at home	91 (80.5)	22 (19.5)	0.79 (0.36, 1.74)		
Varies by family member	70 (66.5)	35 (33.5)	1.65 (0.87, 3.14)		
Perceived COVID-19 risk					
Not concerned/ little concerned	83 (74.3)	29 (25.7)	Ref.		
Concerned/very concerned	850 (76.9)	255 (23.1)	0.87 (0.44, 1.70)		
Social support scale					
Social support index					
Neutral/Disagree/Strongly disagree	101 (53.8)	87 (46.2)	Ref.	Ref.	Ref.
Strongly agree/ Agree	833 (80.9)	197 (19.1)	0.28 (0.17, 0.45)***	0.29 (0.18, 0.48)***	0.29 (0.18, 0.47)***
Scale of pandemic-related difficulties (Autonomy)					
How much control do you have over decisions about leaving the house to go into the community?					
Some/None	518 (77.9)	147 (22.1)	Ref.		
Full control	415 (75.2)	137 (24.8)	1.16 (0.79, 1.72)		
How much control do you have over decisions about seeking health care?					
Some/None	427 (79.3)	111 (20.7)	Ref.		
Full control	507 (74.6)	172 (25.4)	1.30 (0.87, 1.96)		
How much control do you have over decisions about who you will associate with outside of your household?					
Some/None	399 (81.6)	90 (18.4)	**Ref.**	Ref.	**Ref.**
Full control	535 (73.4)	194 (26.6)	1.60 (1.05, 2.44)*	1.51 (0.98, 2.31)	1.52 (1.00, 2.33)*
How much control do you have over decisions about how to spend money you have earned?					
Some/None	632 (78.7)	171 (21.3)	Ref.		
Full control	302 (72.8)	113 (27.2)	1.38 (0.93, 2.05)		
Intimate partner violence/Sexual violence^£^					
In the past 12 months, has a partner ever pushed you, thrown something at you that could hurt you, punched or slapped you?					
Never	289 (76.1)	91 (23.9)		**Ref.**	-
At least once	26 (44.5)	32 (55.5)		3.44 (1.64, 7.21)***	-
In the past 12 months, have you had sex with a partner when you did not want to due to threats, pressure, or force?					
Never	295 (73.6)	106 (26.4)		Ref.	
At least once	20 (53.9)	17 (46.1)		2.38 (0.93, 6.08)	
In the past 12 months, have you had sex when you did not want to with anyone else (not a partner) due to threats, pressure or force?					
Never	305 (72.8)	114 (27.2)		Ref	
At least once	10 (51.2)	9 (48.8)		2.56 (0.61, 10.73)	
Financial situation					
Contributor of the majority of the household income					
Partner/spouse and other contributions	141 (68.7)	64 (31.3)	Ref.		
Respondent	290 (78.4)	80 (21.6)	0.72 (0.31, 1.68)		
Dad or father figure /Mom or mother figure	502 (78.3)	139 (21.7)	0.59 (0.3, 1.17)		
Changes in the respondents’ household responsibilities since COVID-19 restrictions					
No change	286 (81.3)	66 (18.7)	Ref.		
Decreased somewhat/a lot	83 (68.6)	38 (31.4)	2.25 (0.85, 5.98)		
Increased somewhat/ a lot	564 (75.8)	180 (24.2)	2.04 (0.98, 4.24)		
How able the respondent is to meet their basic needs since the COVID-19 restrictions					
Somewhat/ very able	494 (81.1)	115 (18.9)	**Ref.**	Ref.	Ref.
Not very/not at all able	439 (72.3)	168 (27.7)	1.85 (1.02, 3.37)*	1.15 (0.76, 1.74)	1.17 (0.77, 1.77)
Household member laid off of work since start of COVID restrictions					
No	492 (77.6)	142 (22.4)	Ref.		
Yes	217 (72.6)	82 (27.4)	1.22 (0.66, 2.28)		
Worked reduced hours since start of COVID restrictions					
No	443 (82.5)	94 (17.5)	**Ref.**	**Ref.**	**Ref.**
Yes	266 (67.1)	130 (32.9)	2.20 (1.23, 3.96)***	1.86 (1.21, 2.85)**	1.87 (1.22, 2.87)**
Been unable to find work since start of COVID restrictions					
No	508 (80.5)	123 (19.5)	Ref.		
Yes	200 (66.5)	101 (33.5)	1.58 (0.86, 2.91)		
Been unable to work due to illness since start of COVID restrictions					
No	688 (76.8)	208 (23.2)	Ref.		
Yes	21 (55.6)	16 (44.4)	1.76 (0.46, 6.66)		
Been on unpaid leave since start of COVID restrictions					
No	632 (76.7)	192 (23.3)	Ref.		
Yes	77 (70.4)	32 (29.6)	0.73 (0.29, 1.83)		
Stopped attending school since start of COVID restrictions					
No	303 (74.7)	103 (25.3)	Ref.		
Yes	405 (77)	121 (23)	0.88 (0.49, 1.59)		
Gone without meals/ reduced means since start of COVID restrictions					
No	586 (81.1)	137 (18.9)	**Ref.**	**Ref.**	**Ref.**
Yes	123 (58.6)	87 (41.4)	2.58 (1.33, 5.04)**	1.75 (1.04, 2.94)*	1.90 (1.13, 3.20)*

N, weighted sample; %, weighted percent; 95%CI, 95% confidence interval; *p < 0.05; **p < 0.01; ***p < 0.001. The bold values are statistically significant.

From the results of the multivariable analysis, the factors that were significantly associated with having depressive symptoms among all young people included: main activity before COVID-19, social support, having worked reduced hours since start of COVID restrictions and having gone without meals/reduced means since start of COVID restrictions. Control over decisions about who to associate with outside of the household had borderline significance. Respondents who worked reduced hours since start of COVID restrictions (aOR = 2.03; 95% CI: 1.25, 3.28), those who reported having full control over decisions about who they associate with outside of their household (aOR = 1.52; 95% CI: 1.00, 2.33) as well as those who reported having gone without meals/reduced means since start of COVID restrictions (aOR = 1.90; 95% CI: 1.13, 3.20) had significantly higher odds of having depressive symptoms (*p* < 0.05). Young women who, in the past 12 months, had a partner ever pushed them, thrown something at them that could hurt them, punched, or slapped them (aOR = 3.44; 95% CI: 1.64, 7.21) had significantly higher odds of having depressive symptoms (*p* < 0.05). On the other hand, respondents who reported that they can get the emotional help and support they may need from people in their life had significantly reduced odds of having depressive symptoms (aOR = 0.29; 95% CI: 0.18, 0.47).

### Qualitative findings

Several themes surrounding mental health during the onset of the COVID-19 pandemic emerged from the data generated from young men, women, key informants, and stakeholders including: types of mental health problems; contributing factors to mental health problems; and unmet needs for mental health services. Contributing factors to mental health problems comprised loss of work either formal or informal; social isolation; stress over loss of income; and idleness leading to negative behaviors.

### Types of mental health problems

#### Depression and anxiety

Young men and women alike reported adverse mental health experiences as a result of financial and social hardships related to the COVID-19 pandemic. Feelings of depression and stress among youth were attributed to job loss and financial constraints (compounded by subsequent idleness in the home) due to COVID-19.

*I think due to this lack of job opportunities and the way people have lost their jobs, man it has led to depression. You are there and there are people who want to eat, I mean they depend on you … I think most of the people are getting depressed… I mean till you are losing it, you are really losing it you don’t know what you will do.* – 17-year-old female FGD participant

*I mean till you are losing it, you are really losing it, you don’t know what you will do, there is no job. Right now, it is very rare to get job opportunity. There isn’t because everybody is like crying Covid, Covid so I think depression mostly.”* – 19-year-old female FGD participant

*You could find people now becoming stressed, there is a lot of depression because they are social beings so when there is no mingling, people not meeting you are just at home, you are idle, so they became depressed. The relationships were severely affected due to not been able to meet. -* Female Governmental SRH expert

### Contributing factors to mental health problems

#### Loss of work and income

Loss of work, either formal or informal was seen to be associated with several negative consequences, such as loss of hope, increased peer pressure, and substance abuse as aptly described in the following quotes.

*So, you find people like those were used to sell things in the locality, they close such work because it is not bringing much money. Then there it requires they go to…they look for something else and right now you boys there is that phrase they fear…that phrase peer pressure, you find other boys encouraging you to join things like stealing, you use drugs, so you find your life changes, it changes suddenly because of whatever…because of peer pressure in this second of Corona.* - 18-year-old male FGD participant

*Aaa, you may find even that in some families both the breadwinners have lost their jobs, so it is really difficult for them to go on to push on during this tough moment and plus and as it is going you do not see like Covid- 19 is coming to an end because numbers they rise every day. You see, there is no hope people are really suffering a lot things have change and seems there is no way they are going to get out this but I will just hope for the best, you know.* – 15-year-old male FGD participant

*Yeah. Another experience has been stress. Aah, stress caused by lack of money…Stress, caused by idleness…Aah, hopelessness” Aaah (Thinking) most of them of course if you have no money so you get into depression some of them have gone into starting small businesses using their skills to come up with something to bring food on the table. I mean mental health, everybody complained about mental health, the lockdown people don’t want to stay indoors, you have lost your job, maybe you are married and you are there at home, you are not providing for family. So, there is also the aspect of the mental health issues that maybe were not as much as they are right now.* – Female governmental gender expert

A stakeholder further observed that mental health challenges may be due to youth seeing how their parents are struggling to make ends meet.

*So, and also at household level, you know that many have seen the way their parents are struggling now to make eeh to make ends meet. And that of course is equally giving them a lot of mental health challenges.* – Male youth family planning officer

#### Idle time and its consequences

Throughout interviews, idle time and how the youth and adolescents were coping with such idle time were mentioned as challenges. Specifically, young people were not prepared on how to use the extra time they had and sometimes idle time was spent by engaging in activities such drug use or joining groups that model poor behaviors.

*Without money by the way we can’t make because anyway we need something that will us on moving you find at the moment so many youths are idle right now there is nothing so many youths we are engaging with drugs things like such bad things, you see?* – 20-year-old male FGD participant

*Okay because we don’t have any much of responsibilities both in the house or both in the academic sector, because the only thing we have is whatever, our own revision, revision and whatever, and homework we were given in school so you find that boys engaging in like taking drugs, you see, bad company and stuff. So that’s really mostly affecting boys, because you find that the boys are idle they are in the house may be he has finished house chore instructions he has decided let me go take a walk outside, let me go see things and stuff then he meets with some bad boys then he ends up, you know falling into bad behaviors, immoral activities, taking drugs, and it will trickle down up to.* – 15-year- old male FGD participant

A key informant corroborated youth narratives, stating because of the idle time, individuals may engage in criminal activities and substance abuse, thereby increasing insecurity in the community.

#### Social isolation

Being restricted to homes and lack of social gatherings also caused challenges for adolescents and youth. They described lost opportunities to socialize and spend time with their partners, as well as the breakdown of relationships and lack of safe places as summarized in the following quotes.

*Aaa, what I can say on how this pandemic has affected our relationship eh, you find that you cannot be able to see members of your family may be people are not living […] it has really affected all of us our way of life.* – 17-year-old male FGD participant

*First I have, I’ve missed some of my friends, that’s one. Aah then also, then also keeping away people that, that you love, you know. It’s not; it’s not easy so that one is also a barrier to me. …Aah for the last two months, I was not that able much because I was mostly using the social media, yeah. And you know the expenses of social media so that could not allow me to always contact them regularly.* 24-year-old male IDI participant

*Stressors, also being together with my friends. You know right now you cannot stay at one place because of social distancing.* – 23-year-old male IDI participant

*Look at now the young people most of them they like that ah, mm, social with their friends, with their peers, so they are now able to go then they are being restricted to stay at home or even if they go, there is no social gathering. So that case, they are not even able to ah, mm…talk freely; that safe space for them, there is no safe space for the young people, be it at home it be it outside.* – 35-40-year-old male youth advocate

#### Other stressors

Adolescents and youth expressed concerns about the disruption of future plans caused by COVID-19.

*There are disappointments among the youth…because people had disappointments because…Most people had other plans maybe some they will go to campus. Or they will do that, now people are disappointed, they lost hope they engage in inaudible. Covid 19 has also affected the mental health of many many many of us. Because most people lost their jobs. Others had their plans and their plans had, bumped on a rock. So they don’t even know what to do. So, mental health has really really gone. – 17-year-old female FGD participant*

*Yes you find someone is distressed. You get someone is having pressure as in they were expecting good news come 2020. They had set targets then they just raise up and realize that everything has bounced that is you just remain there. You are stranded you cannot do anything you are under somebody.* – 19-year-old male IDI participant

### Unmet need for mental health services

Young people, as well as key informants and stakeholders, reported that there are mental health challenges (i.e., psychological challenges or stress), noting that the “greatest need at the time of the survey” was psychological support. It was suggested that family members require counseling on how to cope with the challenges emanating or associated with COVID-19.

*Yes. At the moment the greatest need according to me for youth now is psychological support. At the moment the way conditions are now considering COVID-19. The way status of the family is, you see how status has been, the way poverty has started to reign in the family lets just say family status has changed. So, they see as if there is no other solutions on the problems they have now. So right now they need counseling. How they will handle these stress they have at the moment, they see in the family mother is not providing, father is not providing. So, they just need support just to be counseled.* – 17-year-old male FGD participant

*And of course, now depression has come in…Because their parents, their guardians, their bread winners, there is a lot of mental health issues moving on and it is becoming a big challenge among young people. They might need a lot psychological support just to ensure that they are able to maneuver through this pandemic safely. So mental health has also become a very very big problem among the young people.* – Female Governmental SRH expert

*Yes. At the moment the greatest need according to me for youth now is psychological support. At the moment the way conditions are now considering COVID-19. And second is counselling. Most young people need counselling.* – 30-35-year-old male Community Health Volunteer

*... There is a lot of psychosocial support that is required for young people. And even as we continue to improve, to open the economy We might still need to lift the livelihoods of young people. How we are going to do that? I don’t know, I don’t know how we are going to do that maybe engaging "partners to support young people.* – 35-40-year-old male Sexual and Reproductive Health/HIV Officer

## Discussion

Findings from this study show that more than one in four adolescents and young men and women aged 15–24 years experienced symptoms consistent with depression. We also found that 10.7% young women and 6.3% young men felt hopelessness nearly every day. Depressive symptoms were associated with reduced working hours due to COVID-19 and increased intimate partner violence. Individuals who reported having special support were less likely to have depressive symptoms. Qualitative results support the quantitative findings and illustrate the negative impact of COVID-19 prevention measures on youth mental health.

Our study found that prevalence of depression among young men was 27%, while it was 30% for young women. These estimates are within the range reported by others (34–36), however, slightly lower than Wu et al.’s systematic review and metanalysis depression prevalence of 34.8% (16.4–55.9) among students (34). A phone-based survey among 10–19 year-olds in Kenya similarly found that 36% of adolescents reported depressive symptoms, and these symptoms were related to food insecurity as a result of COVID-19 (36). There are many determinants of depressive symptoms including age, gender, pre-existing chronic conditions, such as pre-existing anxiety or depression, and even pregnancy status (37–39). Notably, this study was unable to verify whether the participants had conditions which could have worsened their mental health status due to COVID-19.

Results from this study show increased risk of depression for adolescents and young women and men due to idleness and social isolation. Adherence to COVID-19 preventive measures have been previously reported to be a significant life stressor. Dyer et al. found COVID-19-related stressors (e.g., skipping a meal due to not enough money for food, experiencing anxiety, family fighting, and problems in school) were significantly related to adolescent mental health challenges, as measured by suicidal ideation, suicide attempt, and depression (40). Stressful life events such as family conflict (41), problems in school (42), and family fighting (43), are further risk factors for adolescent mental health challenges.

Due to COVID-19 preventive measures, people were confined to their homes, resulting in reduced working time and minimal contact with others outside of immediate family members. Schools were also closed which reduced social contacts with other students (44). While some studies have indicated lost the opportunities to socialize and spend time with their emotional partners and led to breakdowns of relationships (45, 46), others report that social isolation catalyzed new relationships quickly and sparked new or intensified violence in existing relationships (46). We similarly found in our study that depression was associated with intimate partner violence for young women. Notably, given the cross-sectional nature of the survey, we cannot disentangle temporality of intimate partner violence and depressive symptoms.

We also found that the availability of social or emotional support was protective. Indeed, studies have found that parental and social support have better mental health outcomes for young people (47–50); however, it is notable that over 90% of adolescents and young men and women included in this study reported having social support. Longest and Kang study among young adults in US emphasized emotional support is critical in reducing depressive symptoms, especially having some who shows love and listens (50). Social cohesion has been described at the antidote to health anxiety and stress (51, 52).

## Study limitations

Interpretation of study findings need to consider some limitations. First, we used PHQ-2 to quantify mental health, instead of PHQ-9. While the PHQ-2 has been shown to be reliable in assessing depressive symptoms (26, 53), it may not thoroughly cover all depressive symptoms. We did not screen for pre-existing conditions which could have worsened the impact on COVID-19 on the participant’s mental health status (54). However, prevalence reported is similar to those generated from systemic reviews and metanalysis (37). The study relied on self-reported information, which was not verified, though the qualitative data supported the quantitative results. The cross-sectional design limited the possibility of examining causal relationships. Moreover, this is a secondary analysis of mental health needs within a larger project scope; as such, our ability to thoroughly examine mental health needs quantitatively and qualitatively is limited—future studies should aim to focus specifically on mental health needs of adolescents and. Despite these limitations, current findings highlight the magnitude of mental health challenges among adolescents and youth in a setting where mental health services are not readily accessible.

## Conclusion

Adolescents and young persons aged 15 to 24 years comprise about 20% of Kenya’s population. Our study shows that a high proportion of young men and young women experience symptoms of depression, and many of these may be related to COVID-19. Like in many LMICs, mental health needs of the population in general, as well as adolescents, have generally received very little attention. In Kenya, current mental health specific policies do not target the needs of this age group. Prior to COVID-19, there was a need to provide mental health services to adolescents since about 50% of mental health challenges start by age 14 (5, 55); with the onset of COVID-19, it has become critical for services to match these needs. Our findings on the protective effect of emotional and social support highlight a need for families, peers, and communities to support adolescents in order to mitigate the psychological effect of COVID-19 pandemic. Our findings suggest a need prioritize psychosocial programming for adolescents in community and school-based settings to ensure that all adolescents receive appropriate and mitigate long term consequences of mental health challenges later in life (8, 55, 56). Integration of mental health services within healthcare settings can further mitigate long-term impact, while aiming to destigmatize seeking care for mental health needs.

## Open access

This is an open access article distributed in accordance with the Creative Commons Attribution 4.0 Unported (CC BY 4.0) license, which permits others to copy, redistribute, remix, transform and build upon this work for any purpose, provided the original work is properly cited, a link to the licence is given, and indication of whether changes were made. See: https://creativecommons.org/licenses/by/4.0/.

## Data availability statement

The datasets presented in this study can be found in online repositories. The names of the repository/repositories and accession number(s) can be found at: pmadata.org.

## Ethics statement

The studies involving humans were approved by Kenyatta National Hospital-University of Nairobi Scientific and Ethics Review Committee and the Institutional Review Board (IRB) at the Johns Hopkins Bloomberg School of Public Health. The studies were conducted in accordance with the local legislation and institutional requirements. The participants provided their oral informed consent to participate in this study.

## Author contributions

PG, MD, MB, and MT: study design and conceptualization. PG, MB, and MT: protocol development. PG, MT, MW, EG, and PA: analysis. PG, MD, BD, and MB: writing. PG, MT, EG, PA, SW, and MD: editing and interpretation of data. All authors contributed to the article and approved the submitted version.

## Funding

This work was supported, in whole, by the Bill & Melinda Gates Foundation [010481]. Under the grant conditions of the Foundation, a Creative Commons Attribution 4.0 Generic License has already been assigned to the Author Accepted Manuscript version that might arise from this submission. The funders had no role in the study design, collection, analysis, and interpretation of data, in writing of the report, or in the decision to submit for publication.

## Conflict of interest

The authors declare that the research was conducted in the absence of any commercial or financial relationships that could be construed as a potential conflict of interest.

## Publisher’s note

All claims expressed in this article are solely those of the authors and do not necessarily represent those of their affiliated organizations, or those of the publisher, the editors and the reviewers. Any product that may be evaluated in this article, or claim that may be made by its manufacturer, is not guaranteed or endorsed by the publisher.
